# Rhizosphere Diazotrophs and Other Bacteria Associated with Native and Encroaching Legumes in the Succulent Karoo Biome in South Africa

**DOI:** 10.3390/microorganisms10020216

**Published:** 2022-01-20

**Authors:** Esther K. Muema, Emma T. Steenkamp, Stephanus N. Venter

**Affiliations:** DSI-NRF Centre of Excellence in Plant Health Biotechnology, Department of Biochemistry, Genetics and Microbiology, Forestry and Agricultural Biotechnology Institute, University of Pretoria, Pretoria 0002, South Africa; emma.steenkamp@fabi.up.ac.za (E.T.S.); fanus.venter@fabi.up.ac.za (S.N.V.)

**Keywords:** Succulent Karoo Biome, legumes species, diazotrophs, metabarcoding, rhizosphere soils, 16S rRNA gene, *nifH* gene

## Abstract

Total and diazotrophic bacteria were assessed in the rhizosphere soils of native and encroaching legumes growing in the Succulent Karoo Biome (SKB), South Africa. These were *Calobota sericea*, *Lessertia diffusa*, *Vachellia karroo*, and *Wiborgia monoptera*, of Fabaceae family near Springbok (Northern Cape Province) and neighboring refugia of the Fynbos biome for *C. sericea* for comparison purposes. Metabarcoding approach using 16S rRNA gene revealed *Actinobacteria* (26.7%)*, Proteobacteria* (23.6%)*, Planctomycetes,* and *Acidobacteria* (10%), while the *nifH* gene revealed *Proteobacteria* (70.3%) and *Cyanobacteria* (29.5%) of the total sequences recovered as the dominant phyla. Some of the diazotrophs measured were assigned to families; *Phyllobacteriaceae* (39%) and *Nostocaceae* (24.4%) (all legumes), *Rhodospirillaceae* (7.9%), *Bradyrhizobiaceae* (4.6%) and *Methylobacteriaceae* (3%) (*C. sericea*, *V. karroo*, *W. monoptera*)*, Rhizobiaceae* (4.2%; *C. sericea, L. diffusa, V. Karroo*), *Microchaetaceae* (4%; *W. monoptera*, *V. karroo*), *Scytonemataceae* (3.1%; *L. diffusa, W. monoptera*), and *Pseudomonadaceae* (2.7%; *V. karroo*) of the total sequences recovered. These families have the potential to fix the atmospheric nitrogen. While some diazotrophs were specific or shared across several legumes, a member of *Mesorhizobium* species was common in all rhizosphere soils considered. *V. karroo* had statistically significantly higher Alpha and distinct Beta-diversity values, than other legumes, supporting its influence on soil microbes. Overall, this work showed diverse bacteria that support plant life in harsh environments such as the SKB, and shows how they are influenced by legumes.

## 1. Introduction

The Succulent Karoo Biome (SKB) in South Africa is one of the world’s most species rich biomes, with over 5000 plant taxa of which 40% are endemic to the region [1,2,3]. The main vegetation cover includes leaf-succulent, stem-succulent and non-succulent plant species [2,4]. The SKB is semi-arid, experiences winter rainfall, and the high levels of plant diversity have been ascribed to the region’s oscillating wet and dry climatic conditions, moderate climatic history and soils that are sandy, acidic and nutrient limited [4,5].

The need for conservation of this ecosystem has directed some research on the influence of rainfall and droughts on vegetation cover [3,6]. However, the fact that the success and survival of plant species in this relatively harsh environment are due to their interactions with microbial communities in the soil, is often underestimated [7,8].

Members of the Fabaceae family form a significant part of the non-succulent plant species in the SKB, particularly due to their ability to survive in extreme environments [9,10,11,12]. These plants generally possess the ability to fix atmospheric nitrogen in association with symbiotic and free-living soil bacteria [13]. For example, diverse species of *Ensifer* were reported to be the nodulating symbionts of *Vachellia jacquemontii*, thus promoting the plant’s growth in alkaline soils in the Indian Thar Desert [14]. Recent studies that focused on the root-nodule bacteria in the Fynbos biome, neighboring the SKB, revealed that diverse rhizobia (*Azorhizobium*, *Bradyrhizobium*, *Ensifer, Mesorhizobium Rhizobium*, and *Paraburkholderia*) are associated with a wide range of host legumes [15]. However, there is limited information about plant-microbe interactions in the SKB particularly using the next generation sequencing approaches. These include, the post-fire occurrences response of microbial communities using the polymerase chain reaction denaturing gradient gel electrophoresis technique [16] and the association of fungal communities with *Aizoaceae* plants in the Namaqua National Park in the SKB using the metabarcoding approach of the ITS1 region specific primers [8]. Based on sanger sequencing approach, there are some rhizobial interactions with *Vachellia karroo* and *Lessertia* spp. reported [17,18].

Plant-microbe interactions may also account for the establishment and subsequent invasiveness of non-native plants in new areas [19,20]. Such success is attributed to the ability of the invader plant to reconstitute the rhizosphere communities, either through the formation of new associations with the resident microorganisms or by co-introduction with its own interacting partners [19,21]. For instance, many invasive *Acacia* species have been co-introduced with their preferential diazotrophic *Bradyrhizobium* symbionts [22,23]. The consequences of such invasions include loss of native plant diversity and changes in the structure and composition of microbes in the soil [20,24]. This could also be the case for the noted encroachment of *Vachellia karroo* in the SKB from other biomes in South Africa [17,25], which is a topic that has not been addressed previously.

This study explored the diversity and abundance of bacteria associated with different indigenous legume species (i.e., *Calobota sericea*, *Wiborgia monoptera*, *Lessertia diffusa*) and an encroaching *V. karroo* species near Springbok (Northern Cape Province, SA). Microbes sustain ecosystems through nutrient cycling processes. Therefore, understanding plant-microbe interactions is important in the management of the SKB given its significance in the agricultural sector since fodder shrubs such as *C. sericea* support livestock [26]. The sampling sites included those from the understudied arid soils of the SKB and the neighboring refugia of the Fynbos biome, for comparative purposes. Of these legumes, *C. sericea* is commonly distributed in the Northern Cape, Western Cape, and Eastern Cape Provinces of South Africa [26]. *L. diffusa* occurs in the western part of the Northern Cape, in a small part of the Western Cape and North West Provinces [26] while *W. monoptera* occurs across the Greater Cape Floristic Region of South Africa [27,28]. Generally, *C. sericea* is more commonly distributed than *L. diffusa* and *W. monoptera* species. Although *V. karroo* is native to southern Africa, it is considered to be encroaching on the SKB [25].

We utilized a metabarcoding approach based on the 16S ribosomal RNA (rRNA) gene and the *nifH* gene encoding one of the nitrogenase subunits to unveil the total and diazotrophic bacteria in legumes’ rhizosphere soils considered.

## 2. Materials and Methods

### 2.1. Study Sites

**Figure 1:**
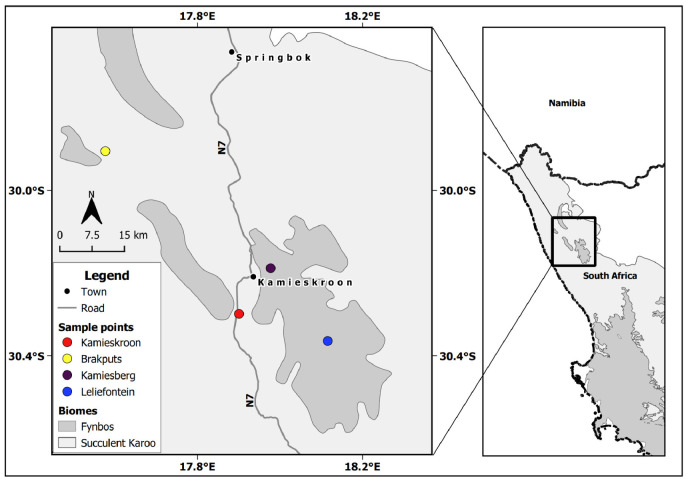
Sampling sites in the Succulent Karoo Biome and the neighbouring patches of the Fynbos biome in the western part of the Northern Cape Province in South Africa.

The SKB of South Africa is situated in the western part of the Northern Cape Province. It lies below 800 m but may reach up to 1500 m above the sea level (a.s.l) on the eastern side. It is characterized by semi-arid to arid conditions, receiving 20 to 290 mm of rainfall per year during the winter season. The temperature may rise beyond 40 °C during the summer periods. SKB soils are generally acidic, weakly developed and mostly found on rocks with limited nutrients [2,5,29]. Based on the occurrence of legumes, two sites within this biome were identified, which were Kamieskroon (30°17′15.74″ S, 17°51′43.81″ E), and Brakputs (29°54′19.43″ S, 17°34′37.41″ E). For comparative purposes, two nearby sites in the Kamiesberg Center, i.e., Leliefontein (30°21′50.09″ S, 18° 6′56.42″ E) and Kamiesberg (30°11′17.52″ S, 17°58′38.67″ E) were also selected (Figure 1). The Kamiesberg Center is an outlying area of the Fynbos, mostly above 1200 m a.s.l., with climatic conditions and soil properties that are generally similar to those of the SKB [30,31].

### 2.2. Soil Sampling and Physico-Chemical Properties

In total, 24 composite soil samples were obtained from the four sites under the four different legumes. Five soil sub-samples were randomly scooped at a depth of 0–10 cm using a soil auger in the root zone area under each legume. The five sub-samples were bulked to form one composite sample per plant. Such samples were collected for *C. sericea* from Brakputs (*n* = 3), Kamiesberg (*n* = 4), Kamieskroon (*n* = 2) and Leliefontein (*n* = 1), while those for *V. karroo* were collected from Brakputs (*n* = 5), and Kamieskroon (*n* = 1). For *W. monoptera* from Brakputs (*n* = 3) and *L. diffusa* from Brakputs (*n* = 5). The composite soil samples were stored at 4 °C. Prior to analysis, each composite soil sample was split into two proportions. The first proportion was sieved (2 mm mesh) and used for soil properties analysis of pH using the KCl method, total carbon (TC), and total nitrogen (TN) using the dry combustion method, ammonium (NH_4_^+^), nitrates (NO_3_^−^) measured calorimetrically and soil texture determined using the Hydrometer method at Bemlab, Cape Town, South Africa. The second proportion was kept at −70 °C for subsequent analyses.

### 2.3. Soil Metabarcoding Analysis

A culture-independent (metabarcoding) approach based on next generation sequencing of specific marker genes was used to investigate the diversity and abundance of total and diazotrophic bacteria in the collected soils [32]. Total soil genomic DNA was isolated from 0.5 g of each composite soil sample using the FastDNA^TM^ SPIN Kit for Soil (MP Biomedicals, Solon, OH, USA). The quality and quantity of extracted DNA were evaluated using 1.0% (*w*/*v*) agarose (Whitehead Scientific, Cape Town, South Africa) gel electrophoresis and a NanoDrop™ 2000/2000c spectrophotometer (Thermo Fisher Scientific, Waltham, MA, USA). For each soil DNA sample, two consistent NanoDrop readings, whose variation was less than 5%, were considered for subsequent determination of the DNA concentration.

The two metabarcoding markers used were the universal house-keeping 16S rRNA gene and the *nifH* gene specific to diazotrophs [33]. The extracted DNA was split into two proportions. The first proportion was sent to the Biomedical Core Research Facilities at the University of Michigan, Ann Arbor, USA for sequencing of the V4 hypervariable region of the 16S rRNA gene. For library preparation, forward and reverse 515F/806R primers contained Illumina-sequencing P5 and P7 dual-indexing adapters with a unique barcode for every sample for multiplexing and demultiplexing [34]. PCR conditions were as follows: 94 °C for 3 min, followed by 33 cycles of 94 °C for 30 s, 55 °C for 30 s and 72 °C for 30 s with a final elongation step at 72 °C for 5 min.

The second DNA proportion was sent to MR DNA (www.mrdnalab.com, accessed on 2 October 2021, Shallowater, TX, USA) for sequencing of the *nifH* gene using PolF/PolR primers [35]. For these reactions, the forward primer contained a barcode. The amplifications were carried out using the HotStarTaq Plus Master Mix Kit (Qiagen, Germantown, MD, USA) with cycling conditions as follows: 94 °C for 3 min, followed by 28 cycles of 94 °C for 30 s, 53 °C for 40 s, and 72 °C for 1 min and a final elongation step at 72 °C for 5 min.

For both genes, amplicons from different samples were pooled together in equal concentrations and purified using calibrated Agencourt Ampure XP beads (Agencourt Bioscience Corporation, Beverly, MA, USA). Paired-end sequencing was performed using the Illumina MiSeq platform to produce paired reads that were 250 nucleotides in length. All raw sequence data for the 16S rRNA and *nifH* genes have been deposited with links to BioProject accession numbers (PRJNA767582, PRJNA767584), respectively.

The 16S rRNA sequence data were processed and analysed using Mothur version 1.35.1 [36]. After joining the paired forward and reverse sequence reads, the sequence quality was checked by specifying a maximum length of 275 base pair (bp) and a minimum of 250 bp, as well as removal of all sequences with ambiguities and with more than 8 bp homopolymers. The sequences were then sorted into their respective samples by allowing of maximum of 2 bp mismatches with the primer and no mismatches in the multiplexing barcodes. The quality-filtered and sorted sequences were then aligned to the SILVA ribosomal RNA reference database [37] provided through Mothur and filtered (filter.seqs option in mothur) to ensure that all the sequences aligned to a similar region of the V4 region of the 16S rRNA gene. These sequences were then processed using a pseudo-single linkage algorithm with the pre.cluster command in Mothur using a 2 bp similarity cut-off [36,38]. In addition, chimeras were detected and removed [39] using the chimera.uchime command in Mothur. The remaining sequences were then associated with particular taxonomic lineages using the Greengenes database [40] with a cut-off confidence level of 80%. After removing sequences of unknown taxonomic lineages, the remaining data were classified into operational taxonomic units (OTUs) using average neighbour algorithm with a similarity cut-off of 97%. OTUs were assigned by obtaining a representative sequence from each OTU (get.oturep command in Mothur) and classifying it using the Greengenes database at a confidence level of 80% [40].

The *nifH* sequence data were processed using the analysis pipeline of MR DNA (www.mrdnalab.com, accessed on 2 October 2021, Shallowater, TX, USA) according to qiime pipeline using the divisive amplicon denoising algorithm (dada2) [41]. This entailed the joining of paired end sequences and quality filtering. For the latter, sequences shorter than 200 bp, containing more than two ambiguous characters, that had homopolymer runs longer than 6 bp, or that contained more than one mismatch to the sample-specific barcode or to the primers sequences were removed. Following removal of the barcode and primer sequences, the raw *nifH* reads were denoised to remove sequencing errors, separated into OTUs defined by clustering at 97% sequence similarity [42] using a curated and publicly available database of *nifH* sequences (https://wwwzehr.pmc.ucsc.edu/, accessed on 15 September 2021) [43], after which chimeras were removed [44,45,46]. Final OTUs were taxonomically classified using BLASTn against this database.

### 2.4. Statistical Analyses of Metabarcoding Data and Soil Physico-Chemical Properties

Prior to statistical analyses, singletons were removed using the filter.shared command in Mothur (version 1.35.1) [36]. Alpha and beta diversity analyses were conducted to compare samples based on OTU assignment. Alpha diversity indices (richness, non-parametric Shannon diversity and Shannon evenness) [47] were calculated using the summary.single command in Mothur [36]. Beta diversity OTU-based (Bray-Curtis and Jaccard) matrices to compare the structure (Bray-Curtis) and membership (Jaccard) of the samples were calculated using the dist.shared command in Mothur [36]. The least number of sequences across all the samples after removal of singletons were set as the sampling depths (i.e., *n* = 1465 for 16S rRNA and *n* = 2691 for *nifH*) and subsampled 1000 times (iters = 1000) to standardize both the alpha and beta diversity calculations. Good coverage estimate for the alpha diversity matrices was used to determine the % coverage within samples after sub-sampling.

Prior to analysis of variance (ANOVA) or permutations analysis of variance (PERMANOVA), a legume rhizosphere single soil sample from Leliefontein was eliminated. Therefore, four legume species (*C. sericea*, *L. diffusa*, *V. karroo*, and *W. monoptera* from three sampling sites (Brakputs, Kamiesberg, and Kamieskroon) totalling to 23 samples were considered. Alpha diversity and soil properties data were subjected to normality tests using histograms and Q-Q plots to check the homogeneity of variance in R (v.4.0.0) software (https://www.rstudio.com, accessed on 5 September 2021). One-way ANOVA was conducted to determine whether the Alpha diversity indices and soil properties were significantly different under legumes and sites where the samples were collected. The Akaike information criterion (AIC) was used to select the best-fit model to explain variation in the dependent variables. Table of *p* values for all possible pairwise comparisons of least square means (lsmeans), and the letters (letter display) were conducted using the Tukey’s honestly significant difference (Tukey’s HSD) post-hoc test in R (v.4.0.0). Pearson correlation and regression analysis with the soil properties were also conducted using R (v.4.0.0) to test if these data were correlated and by how much the properties explained the changes in the alpha diversity indices. PERMANOVA was conducted to test if legume species and sites influenced the Beta diversity matrices.

For graphical visualization of the structure of communities as shaped by the legumes in the respective sampling sites, non-metric multidimensional scaling (NMDS) and principal co-ordinate (PCoA) were performed in R (v.4.0.0) on OTU-based matrices (Bray-Curtis and Jaccard) [48]. Canonical analysis of principal coordinates (CAP) was also conducted in R (v.4.0.0) to emphasize the influence of soil properties on the structure of bacteria [49]. Heatmaps were generated using relative abundance data of OTUs using the R (v.4.0.0) package heatmap.

## 3. Results

### 3.1. Physico-Chemical Properties of the Studied Rhizosphere Soils from the Succulent Karoo Biome

The results of the soil properties analysis are presented in Table SI. Overall, soil properties were not significantly different among the sampling sites (Brakputs, Kamiesberg and Kamieskroon) (*p* > 0.05). Legume species, however, revealed significant differences for pH and TC (*p* < 0.05). Overall, pH values ranged from 4.00 (*W. monoptera*) to 6.47 (*V. karroo*). The pH values associated with *V. karroo* were significantly higher than those from *W. monoptera* and *C. sericea* (*p* < 0.05). The pH therefore ranged from extremely acidic for *W. monoptera*, to strongly acidic for *C. sericea*, *L. diffusa* and slightly acidic for *V. karroo*. TC values ranged from 0.61 to 1.93 (%) for *L. diffusa* and *V*. *karroo*, respectively. TC values under *V. karroo* were significantly higher than those from *C. sericea* and *L. diffusa* (*p* < 0.05). TC values were generally low for all the species, except *V. karroo* where it was high. TN values ranged from 0.05 to 0.16 (%) for rhizosphere soils from *L*. *diffusa* and *V. karroo*, respectively. Generally, TN values ranged from low to very low for all the legume rhizosphere soils analyzed. The NH_4_^+^ and NO_3_^−^ content ranged from 9.46 to 17.1 mg-N kg^−1^ and 0.57 to 10.9 mg-N kg^−1^, respectively. There were no significant differences observed for TN, NH_4_^+^ and NO_3_^−^ values between the different legumes, although *V. karroo* consistently maintained the highest values (*p* > 0.05).

### 3.2. The Dominant Bacterial Communities in the Rhizosphere Soils Considered

**Figure 2:**
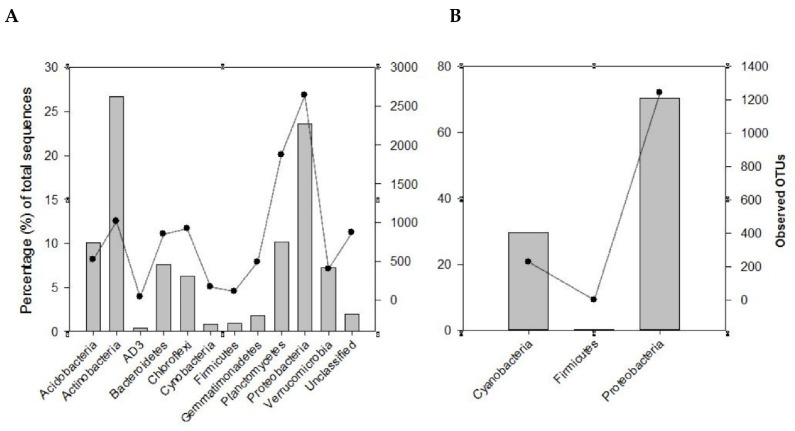
Recovery of the major phyla, using metabarcoding with the 16S rRNA (**A**) and *nifH* (**B**) genes, from the rhizosphere soils of the different legume species from various sites in the study area. The bars represent the percentage total sequences while the lines with a filled circle symbol represent the observed OTUs.

**Figure 3:**
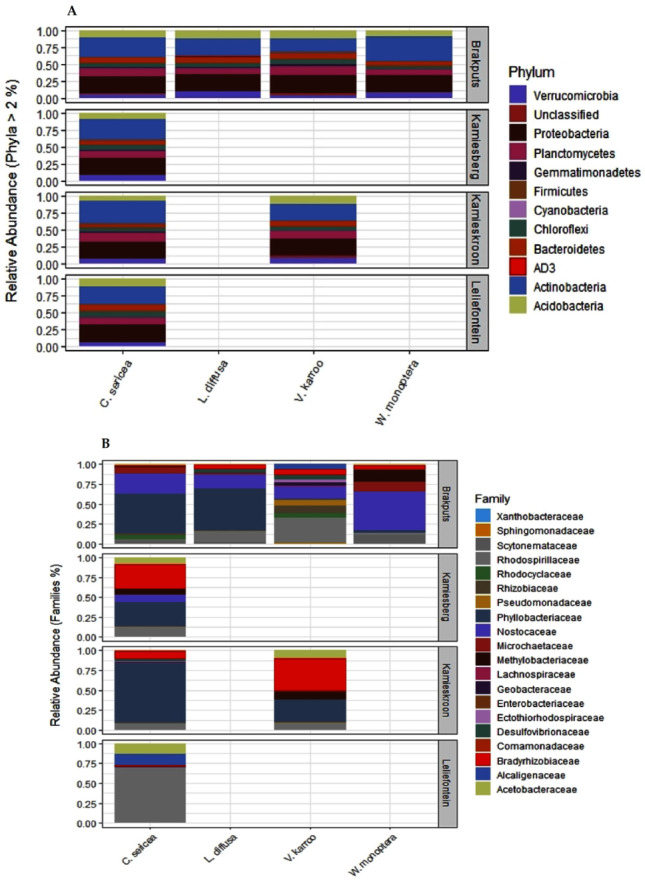
Distribution of relative abundances of microbial communities in the rhizosphere soils of different legume species in the study sites. (**A**) Phylum level relative abundances of total bacteria (16S rRNA), (**B**) relative abundances of dominant nitrogen-fixing families (*nifH* gene).

Using the 16S rRNA metabarcoding, a total of 362,185 sequences were classified into 10,643 OTUs (at 97% similarity threshold) from the 24 rhizosphere soil samples examined. Although good coverage (range 66–91%, average 79%) estimates suggested that dominant taxa would be identified, analysis of a larger volume of sequences is recommended for a comprehensive sampling of the diversity of these communities. The identified OTUs were assigned to biologically and taxonomically diverse species from a range of bacterial phyla (Table S2; Figure 2A). Among these were *Actinobacteria* (26.7%), *Proteobacteria* (23.6%)*, Planctomycetes* and *Acidobacteria* (10%)*, Bacteroidetes* (7.6%)*, Verrucomicrobia* (7.3%)*, Chloroflexi* (6.3%), *Gemmatimonadetes* (1.8%), *Firmicutes* (0.9%), and *Cyanobacteria* (0.8%). These corresponded to 1018, 2641, 1878, 519, 852, 404, 924, 598, 118, and 161 OTUs, respectively (Figure 2A and Figure 3A). These phyla represented approximately 96% and 85% of the total sequences and OTUs observed, respectively. Thirty-eight OTUs representing approximately 18% of the total sequences were shared across all the soil samples from the two biomes. Most of these OTUs belonged to *Actinobacteria* (17 OTUs) and *Proteobacteria* (11 OTUs). Forty OTUs representing approximately 13.6% of the total recovered sequences were shared among the SKB samples. These OTUs were also dominated by *Actinobacteria* and *Proteobacteria*.

Metabarcoding using the *nifH* gene allowed for the classification of 1,094,932 sequences into 1471 OTUs using a similarity threshold of 97%. Good coverage (range 96.4–99.8%, average 99%) indicated that sub-sampling at 2691 sequences covered the majority of the diversity of communities for all the samples. *Proteobacteria* and *Cyanobacteria* (respectively represented by 70.3% and 29.5% of the total sequences) were the most dominant and diverse phyla (Figure 2B). They contained 1241 (84.4%) and 229 (15.6%), respectively, of the total OTUs observed. Overall, these two phyla represented approximately 99.8% and 99.9% respectively, of the *nifH* total sequences and OTUs observed. A small proportion of the sequences (0.20%, 1 OTU) originated from the phylum *Firmicutes*.

Based on the *nifH* data, several diazotrophic families were measured (Figure 3B; Table S3). These consisted of OTUs from the families *Nostoceae, Microchaetaceae* and *Scytonemataceae* (24.4%, 4.0%, and 3.1%), respectively, (*Cyanobacteria*) of the total sequences (Figure 3B; Table S3). Others OTUs belonged to the families *Phyllobacteriaceae* (39.0%), *Rhodospirillaceae* (7.9%), *Bradyrhizobiaceae* (4.6%), *Rhizobiaceae* (4.2%) *Rhodocyclaceae* (3.7%), *Methylobacteriaceae* (3.0%), *Pseudomonadaceae* (2.7%), *Geobacteraceae* (1.78%), *Alcaligenaceae* (1.2%), *Acetobacteraceae* (1.1%), *Desulfovibrionaceae* (0.62%), *Comamonadaceae* (0.18%), *Ectothiorhodospiraceae* (0.16%), *Sphingomonadaceae* (0.12%), and *Enterobacteriaceae* (0.06%) (*Proteobacteria*) of the total sequences. There was also one OTU belonging to the family *Lachnospiraceae* (0.2%) (*Firmicutes*) of the total sequences (Table S3). The high dominance of *Phyllobacteriaceae* was also supported by the recovery of one OTU in all 24 soil samples examined. This OTU represented a member of the genus *Mesorhizobium* and formed 27.3% of the total *nifH* sequences. Two OTUs, members of *Proteobacteria* representing approximately 27.3% of the total sequences were shared among the SKB samples.

### 3.3. OTU-Based Diversity of Diazotrophs as Influenced by Legume Species

Legumes showed common and distinct influences on the diazotrophs at family level in the arid soils of the Succulent Karoo or Fynbos biomes based on *nifH* metabarcoding analysis (Figure 3B; Table S3). For example, the rhizosphere soils of *C. sericea* and *V. karroo* shared most members of the identified families. These included OTUs from *Bradyrhizobiaceae*, *Acetobacteraceae, Phyllobacteriaceae, Rhodospirillaceae, Comamonadaceae, Rhizobiaceae, Rhodocyclaceae, Nostocaceae, Methylobacteriaceae*, and *Sphingomonadaceae*. On the other hand, *L. diffusa* and *W. monoptera* showed few families whose OTUs were mainly shared either with *C. sericea*, *V. karroo* or both. These were OTUs from *Bradyrhizobiaceae*, *Nostocaceae*, *Microchaetaceae, Rhodospirillaceae*, *Methylobacteriaceae*, *Acetobacteraceae*, and *Scytonemataceae* in the rhizosphere soils of *W. monoptera* and OTUs from *Phyllobacteriaceae*, *Rhizobiaceae, Nostocaceae*, and *Scytonemataceae* in the rhizosphere soils of *L. diffusa* (Table S3).

In terms of the association of diazotrophs with specific legumes, the rhizosphere soils of *V. karroo* specifically showed a high diversity and abundance of OTUs representing *Alcaligenaceae, Desulfovibrionaceae, Geobacteraceae, Lachnospiraceae, Ectothiorhodospiraceae and Pseudomonadaceae* among others (Table S3). *Enterobacteriaceae* was specifically found in the rhizosphere of *W. monoptera* (Table S3).

### 3.4. Alpha Diversity

The richness (observed taxa) and structure-based alpha diversity metrics (Shannon Index and Shannoneven Index) were strongly influenced by legumes species (*p* < 0.01) but not the sampling sites or the interactions between legume species and sites (*p* > 0.05) for both 16S rRNA and *nifH* genes (Table S4). Therefore, only the results of the influence of legume species on alpha diversity indices are presented.

Based on the 16S rRNA, *V. karroo* revealed the highest species richness (687 ± 31.6), Shannon index (structure) (6.09 ± 0.08) and Shannoneven index (evenness) (0.932 ± 0.006) (*p* < 0.05) (Table 1). The lowest alpha diversity indices were revealed for *W.* monoptera, species richness (482 ± 44.8), Shannon index (5.45 ± 0.11) and Shannoneven index (0.883 ± 0.008) (*p* < 0.05) (Table 1). Richness was significantly positively correlated with the soil pH, TN and TC (r = 0.70, *p* < 0.001; r = 0.54, *p* < 0.01; r = 0.52, *p* < 0.05) respectively (Table 2). A similar trend was observed for Shannon and Shannoneven indices correlations with soil properties as follows; Shannon index (r = 0.77, *p* < 0.001 (soil pH), r = 0.53, *p* < 0.01 (TN) and r = 0.50, *p* < 0.05) for TC. Shannoneven index (r = 0.79, *p* < 0.001; r = 0.49, *p* < 0.05; r = 0.42, *p* < 0.05) for pH, TN and TC respectively (Table 2). Regression analysis revealed significant effects of soil pH (r^2^ = 0.46; *p* < 0.001), TN (r^2^ = 0.21; *p* < 0.05) and TC (r^2^ = 0.20; *p* ≤ 0.05) for richness. Soil pH (r^2^ = 0.56; *p* < 0.001), TN (r^2^ = 0.22; *p* < 0.01) and TC (r^2^ = 0.18; *p* ≤ 0.05) for Shannon index. Soil pH (r^2^ = 0.59; *p* < 0.001), TN (r^2^ = 0.21; *p* < 0.05) and TC (r^2^ = 0.15; *p* < 0.05) for Shannoneven index (Table 2).

Based on the *nifH*, *V. karroo* revealed significantly higher richness (115.0 ± 12.1) than *C. sericea* (65.5 ± 10.0) and *L. diffusa* (45.2 ± 13.6) (*p* < 0.05) (Table 1). *V. karroo* also had the highest structure (2.91 ± 0.25) while *W. monoptera* revealed the highest evenness (0.67 ± 0.08) (*p* < 0.05) (Table 1). Interestingly, pH did not reveal significant correlations with any of the alpha diversity indices (Table 2). Species richness was significantly positively correlated with TN (r = 0.62; *p* ≤ 0.01), TC (r = 0.66; *p* ≤ 0.001), NH_4_^+^ (r = 0.42; *p* ≤ 0.05) and NO_3_^−^ (r = 0.46; *p* < 0.05) (Table 2). Shannon index was significantly positively correlated with TN and TC (r = 0.55; *p* < 0.01, r = 0.59; *p* < 0.01) respectively, while Shannoneven index was only significantly positively correlated with TC (r = 0.45; *p* < 0.05) (Table 2). Regression analysis revealed significant r^2^ values for TN (r^2^ = 0.38; *p* < 0.01), TC (r^2^ = 0.43; *p* < 0.001), NH_4_^+^ (r^2^ = 0.18; *p* ≤ 0.05) and NO_3_^−^ (r^2^ = 0.21; *p* ≤ 0.05) for richness. Shannon index revealed (r^2^ = 0.30; *p* < 0.01) TN and (r^2^ = 0.35; *p* < 0.01) TC. R^2^ = 0.20; *p* < 0.05 (TC) was revealed for Shannoneven Index (Table 2). 

### 3.5. Beta-Diversity

**Figure 4:**
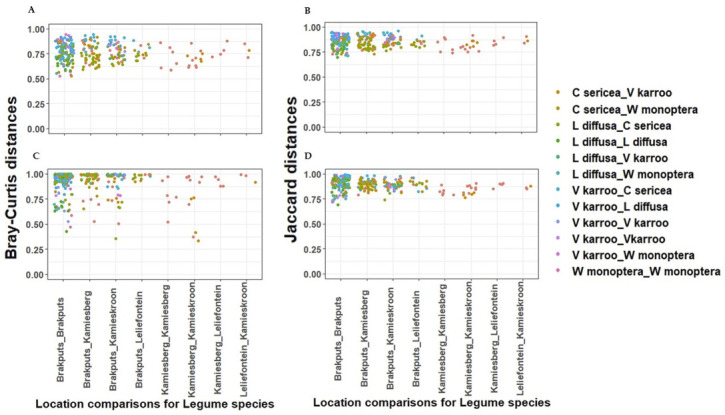
Dissimilarities between sites and legume species using pair wise structure-based Bray-Curtis and Jaccard distances respectively. (**A**,**B**) = 16S rRNA gene; (**C**,**D**) = *nifH* gene.

**Figure 5:**
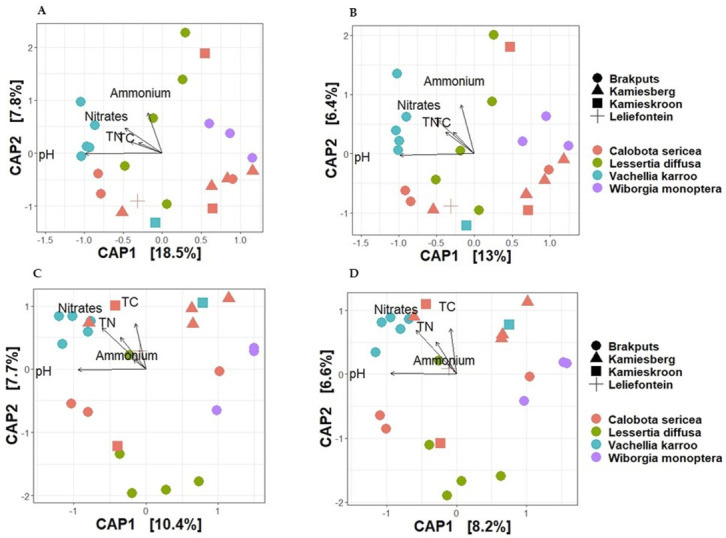
Canonical analysis of principal coordinates (CAP) of Beta-diversity based on Bray-Curtis and Jaccard distances for visual presentations of patterns of composition of bacterial communities with respect to legume species and their relationship with soil chemical properties. (**A**) = 16S rRNA Bray-Curtis, (**B**) = 16S rRNA Jaccard, (**C**) = *nifH* Bray-Curtis and (**D**) = *nifH* Jaccard. TC, total carbon; TN, total nitrogen.

Based on 16S rRNA gene, the average dissimilarity in community structure between legumes and sites was 76 ± 9% (Bray-Curtis), and 85 ± 5% (Jaccard) (Figure 4A,B). These high indices suggested a low level of similarity between the samples examined. Prior to PERMANOVA analysis, ordination analysis (NMDS and PCoA) based on Bray-Curtis and Jaccard matrices revealed legumes specific clustering of bacteria particularly for *V. karroo* (results not shown). PERMANOVA analysis based on both matrices revealed that variability among samples was best explained by the legumes (*p <* 0.001) (Table S4). CAP analysis showed pH, TN, and TC as important factors influencing the 16S rRNA Beta-diversity (Figure 5A,B). For example, communities clustered together in the direction of high pH, TN and TC particularly those associated with *V. karroo*.

The average dissimilarity for the *nifH* gene was 91 ± 13% (Bray-Curtis) and 89 ± 6% (Jaccard) (Figure 4C,D). These indicated a low level of similarity between the samples. Similar to the 16S rRNA, *nifH* gene-based ordination analysis on diazotrophic communities revealed legumes specific clustering particularly for *V. karroo* (Figure 5C,D). PERMANOVA analysis based on both matrices revealed that legumes and sites influenced structure and membership matrices (*p* < 0.01) (Table S4). For example, CAP analysis showed clear clustering patterns among legumes (e.g., *L. diffusa*, *W. monoptera* and *V. karroo*). In addition, nitrates and TN were found as the environmental properties that mainly influenced the *nifH* Beta diversity, as communities clustered in the direction of high nitrates and TN particularly those associated with *V. karroo* and some *C. sericea* species (Figure 5C,D).

## 4. Discussion

### 4.1. Bacteria Composition in Rhizosphere Soils Used Is Associated with Environmental Conditions

Based on the metabarcoding of the 16S rRNA, several of the taxa identified (e.g., *Proteobacteria*, *Actinobacteria*, *Bacteroidetes*, and *Cyanobacteria*) have previously been shown to dominate rhizosphere and desert soils [7,50]. Most of them are reported to have inherent properties making them well suited to the SKB soils, such as adaptation to high temperatures and soil acidity as is the case for the SKB. For instance, many species of *Cyanobacteria, Planctomycetes* and *Verrucomicrobia* are known to thrive in higher temperatures and are tolerant to low soil pH [51,52,53].

The consistent dominance of the *Proteobacteria* phylum using both 16S rRNA and *nifH* genes in this study was in agreement with several other studies which reported their abundance in different soil ecosystems such as agricultural, forests, grasslands, saline as well as semi-arid soils [54]. This is attributed to their role in sustaining these environments through various biogeochemical processes. *Cyanobacteria* members are aquatic, but have also consistently been reported in soil crusts and rhizosphere soils in arid and semi-arid environments, particularly due to their role in atmospheric nitrogen fixation [51,54]. This was consistent with the *nifH* findings in this study. Their low measurements according to the universal 16S rRNA gene could be due to its low-resolution power compared to functional and group specific primers such as the *nifH* gene [55]. *Firmicutes* on the other hand, were less diverse and abundant in the considered rhizosphere soils using the both metabarcoding approaches. This was not surprising as members of this phylum are mainly associated with contaminated environments such as sludges and soil impacted by acid mine drainage [54,56].

Environmental factors strongly influenced the total and diazotrophic bacteria in the soils examined. This was particularly evident for soil pH and nutrients. Significantly positive correlations of >70% for pH and >40% for TN and TC, were observed with the richness and structure of total bacteria. These factors accounted for much of the variation within communities (at least 45% for pH, 20% for TN and at least 15% for TC). These factors further influenced the diazotrophs, as significant positive correlations of >40% were observed for species richness for all the soil properties except pH. TC and TN influenced the structure of the diazotrophs as >55% positive correlations were observed for the species structure, while TC showed 45% positive correlations with species evenness. These factors accounted for at least 18% of variations within the diazotrophs. The influence of environmental factors on microbes was also revealed for beta-diversity analyses. Similar effects of pH, TN, TC, and NO_3_^−^ on soil bacteria were found in the bulk or rhizosphere soils of *Acacia dealbata* in the grassland biome in South Africa [57], and in an agricultural soil in Kenya [58]. Soil pH has also been reported as a main driver of changes in the structure and composition of total soil bacteria [58,59].

It is likely that the different groups of bacteria identified in this study form part of specialized communities, some of which interact with plants to facilitate or enhance their ability to colonize and become established in the harsh environmental conditions of the SKB. Ways in which this facilitation can occur is through nitrogen, carbon and phosphorus compounds cycling, thereby improving the plants’ access to nutrients [60,61]. Many species of the *Gemmatimonadetes*, *Cyanobacteria*, *Proteobacteria*, *Chloroflexi*, *Firmicutes*, and *Acidobacteria*, for example, are known to facilitate carbon assimilation via (bacterio) chlorophyll-based photosynthesis [62,63], while certain *Proteobacteria* and *Firmicutes* are capable of phosphorus solubilization, thus making this element available to plants [64,65]. However, in the nitrogen-poor soils of the SKB, cycling of this element is perhaps equally important, and various members of the *Proteobacteria* [66,67], *Planctomycetes* [53], *Bacteroidetes* [68], *Verrucomicrobia* [69], *Firmicutes* [64], and *Cyanobacteria* [51,52] are known to be capable of fixing atmospheric nitrogen to provide ammonium and/or nitrates for plant growth.

The bacteria in the rhizosphere soils of the legumes investigated are involved in the promotion of the plants’ growth and might even protect them against pathogens. Many *Acidobacteria*, *Firmicutes* and *Proteobacteria* are well known to produce indole-3-acetic acids (IAAs), siderophores and the enzyme aminocyclopropane carboxylate (ACC) deaminase [64,70]. IAA is a plant growth hormone [13,64], and siderophores chelate iron and avail it to plants for growth, while making it unavailable to other organisms, particularly pathogens [64,71]. Under stressful conditions (e.g., drought and heat), the ACC deaminase promotes root growth by lowering ethylene levels in plants [68,71,72,73]. Taken together, the diverse bacteria measured thus underscore the crucial role they play in supporting plants in the SKB.

### 4.2. Legume Species Influence Diazotrophs in the Succulent Karoo Biome

Metabarcoding with the *nifH* allowed a more in-depth analysis of the diazotrophic taxa occurring in the rhizosphere soils examined. Our findings revealed diverse OTUs which were separated into at least 19 families of diazotrophs. The distribution, diversity and abundance of these diazotrophs were however, modulated by legumes considered. For example, the rhizosphere soils of *L. diffusa* were mainly dominated by OTUs belonging to *Phyllobacteriaceae*, *Rhizobiaceae, Nostocaceae*, and *Scytonemataceae*. These families contain species known to fix atmospheric nitrogen. For example, *Phyllobacteriaceae* contained the rhizobial genus *Mesorhizobium* and *Rhizobiaceae* contained the genera *Sinorhizobium*/*Ensifer* among others. These findings support previous reports which demonstrated the symbiotic relationships of *Mesorhizobium* and *Sinorhizobium*/*Ensifer* with *Lessertia* species [17,66]. *Nostocaceae* and *Scytonemataceae* on the other hand, are known to contain species with free-living nitrogen fixation abilities [51]. The rhizosphere soils of *W. monoptera* mainly contained species belonging to *Bradyrhizobiaceae, Methylobacteriaceae*, *Nostocaceae, Rhodospirillaceae, Acetobacteraceae, Microchaetaceae, Scytonemataceae*, and *Enterobacteriaceae* families. *Bradyrhizobiaceae* and *Methylobacteriaceae* members have symbiotic rhizobial capabilities with these legumes, which is consistent with previous reports regarding the distribution of *Bradyrhizobium* and its association with a wide range of legumes [17,28,66]. *Methylobacteriaceae* members have also been reported to possess symbiotic associations with diverse legumes in the papilionoid tribe *Crotalarieae* [74,75]. Members from the other families contain free-nitrogen fixation capacities. For example, members from *Rhodospirillaceae* [76,77] and *Microchaetaceae* [51] as well as *Enterobacteriaceae* that occurred specifically in rhizosphere soils of *W. monoptera* [78]. Moreover, some diazotrophic members belonging to *Acetobacteraceae* promote plant growth potentially through phosphorus solubilization and antagonism against plant pathogens [65]. The rhizosphere soils of *L. diffusa* and *W. monoptera* therefore, contained diazotrophic communities with the ability to mainly fix atmospheric nitrogen and solubilize phosphorus for *W. monoptera* species. According to the alpha-diversity analyses, generally low species richness, structure and evenness was measured under the rhizospheres of *W. monoptera* and *L. diffusa*. Consistently, the communities under these two legumes clustered separately, being distinct from those under *C. sericea* and *V. karroo*. Both legumes are not widely distributed in the SKB, which explains the low diversity, abundance and specialization of their associated diazotrophs.

The rhizosphere of *C. sericea* was dominated by OTUs from *Bradyrhizobiaceae*, *Rhodocyclaceae*, *Rhizobiaceae*, *Nostocaceae*, *Phyllobacteriaceae*, *Rhodospirillaceae*, *Acetobacteraceae*, *Sphingomonadaceae*, *Methylobacteriaceae*, and *Comamonadaceae* families. Species from *Bradyrhizobiaceae*, *Rhizobiaceae*, *Methylobacteriaceae* and *Phyllobacteriaceae* have symbiotic characteristics with legumes in the papilionoid tribe *Crotalarieae* [66,79,80,81]. Members of the *Rhodocyclaceae*, *Nostocaceae*, *Rhodospirillaceae*, *Acetobacteraceae*, and *Comamonadaceae* are free-living nitrogen fixers [82,83,84,85]. In addition, some members from *Acetobacteraceae* are known to promote plant growth through phosphorus solubilization and antagonism against pathogens [65]. Some members from *Comamonadaceae* promote plant growth through carbon cycling [86], while members from *Sphingomonadaceae* are known to promote plant growth through the production of IAAs [87]. These communities did not have specific clustering pattern with *C. sericea* according to the ordination analysis. Therefore, the diazotrophs associated with *C. sericea* are not only diverse and abundant, but also possess different plant growth promoting properties, ranging from atmospheric nitrogen fixation, phosphorus solubilization, production of IAAs and antagonism against pathogens. This could be the reason this legume is commonly distributed in the SKB and also in the neighboring Fynbos biome.

*V. karroo* species had higher diversity and abundance of diazotrophs than all the other legumes. Significantly higher species richness and diversity under *V. karroo* were observed using 16S rRNA-based metabarcoding. These were supported by ordination analysis, where beta-diversity indices showed clustering of members associated with *V. karroo* away from those associated with the other legumes. Additionally, higher pH, TC, TN, NH_4_^+^, and NO_3_^−^ were measured in the *V. karroo* rhizosphere soils than for other legumes. Increased levels of nutrients and pH associated with *V. karroo* are believed to support its ability to attract diverse and abundant bacteria [57,59]. This gives it a competitive advantage in modifying the nutrient status of its rhizosphere soils more strongly than other legumes in the same habitat. In *Acacia sensu lato*, such advantages have been suggested to be as a result of nitrogen fixation, organic matter production via root exudates, and leave fall under their canopy [57]. These mechanisms drive the invasiveness of certain legumes [14,19,20] and may also play an important role in the encroaching behavior of *V. karroo* in the SKB.

The bacterial distribution patterns observed in the rhizosphere communities associated with *V. karroo* suggest that it modified its rhizosphere environment, favouring or enhancing the establishment of particular communities [19,20,24,88]. For example, OTUs from *Geobacteraceae*, *Alcaligenaceae*, *Lachnospiraceae*, *Desulfovibrionaceae*, *Ectothiorhodospiraceae*, and *Pseudomonadaceae* taxa were specifically associated with the rhizosphere soils of *V. karroo*. These diazotrophs are free-nitrogen fixers [61,89,90,91,92,93,94,95]. Moreover, some members from *Alcaligenaceae* promote plant growth through production and control of IAAs levels [87,96]. OTUs such as those from *Pseudomonadaceae* promote plant growth through phosphorus solubilization, production of IAAs and siderophores [97,98,99]. Although this study is the first to report on the metabarcoding-based rhizosphere bacteria associated with *V. karroo*, different invasive trees such as *Berberis thunbergii* DC. (Japanese barberry) have been shown to attract different taxa including *Pseudomonadaceae* in their rhizosphere environment [88]. *Acacia dealbata*, an Australian invasive legume in South Africa, mainly attracted members from *Bradyrhizobiaceae* and *Pseudomonadeceae* among others in its rhizosphere [19,57]. Therefore, interactions between *V. karroo* and particular soil bacteria lead to significant changes in the composition of rhizosphere communities. Other free nitrogen-fixers which were associated with *V. Karroo*, but also found in the rhizosphere of the other legumes included members from *Rhodocyclaceae, Nostocaceae, Rhodospirillaceae, Acetobacteraceae, Microchaetaceae, Sphingomonadaceae,* and *Comamonadaceae* families [51,65,77,84,86,87]. In addition, some members from *Acetobacteraceae*, *Sphingomonadaceae,* and *Comamonadaceae* also promote plants growth through phosphorus solubilization, the production of IAAs, siderophore production for protection against pathogens, and carbon cycling [85,86,100].

With regards to possible rhizobial symbionts, *V. karroo* showed the ability to associate with diverse rhizobia. For example, several OTUs that were affiliated with *Phyllobacteriaceae* (*Mesorhizobium*), *Bradyrhizobium*, and a few with *Rhizobiaceae* (*Rhizobium* and *Ensifer*) formed part of its rhizosphere. These findings are consistent with other studies [14,17,66,101]. Undoubtedly, the ability of *V. karroo* to establish symbiotic interactions with a wide range of rhizobia also provides it with a competitive advantage over other legumes to colonize and encroach on new areas [25]. The high diversity of its potential rhizobial symbionts, combined with unique bacteria in *V. karroo*’s rhizosphere soils in the SKB, support its involvement in reconstructing its microbiome, a strategy used by most invasive members of the mimosoid clade of legumes to invade and colonize new environments [19]. The rhizosphere of *V. karroo* therefore contained the most diverse and abundant diazotrophs that possess a wide range of plant growth promotion properties such as atmospheric nitrogen fixation, phosphorus solubilization, the production of IAAs and siderophores, carbon cycling as well as antagonism effects against pathogens.

Most studies that investigated the influence of plant species and environmental conditions on soil microbial communities have mainly focused on non-legumes versus legumes [55]. For example, legume species *Stylosanthes guianensis* (Aubl.) Sw., *Trifolium pratense* L., and *Medicago sativa* L. enriched soil microbial communities compared to grass species (*Paspalum natatum*, *Festuca arundinacea* L., *Lolium perenne* L.) in China [102]. In Nigeria, a legume species *Pterocarpus erinaceus* improved the soil physico-chemical properties and selected for ten bacterial species compared to a non-legume *Anoigessus leiocarpa*, which was associated with low values of soil properties and only six bacterial species [103]. This study further revealed that legume species, which fall under the same family *Fabaceae*, select for specific microbial communities and also share some microbial communities across their rhizospheres.

## 5. Conclusions

This study revealed diverse diazotrophs associated with legumes in the SKB in South Africa. Environmental factors such as soil pH, soil nutrients, and legume species influenced the microbial communities. A member of *Mesorhizobium* species was common in all rhizosphere soils considered. Other diazotrophs such as *Bradyrhizobiaceae*, *Nostocaceae*, among others were shared across several legume rhizosphere soils, while others such as *Enterobacteriaceae* and *Pseudomonadaceae* were specific to specific legume rhizosphere soils. Legumes such as *L. diffusa* and *W. monoptera* that are not widely distributed in the SKB haboured less diverse and abundant diazotrophs which mainly possess atmospheric nitrogen fixation properties compared to the widely distributed *C. sericea* and the encroaching *V. karroo* species. These legumes both contained diverse diazotrophic communities, associated with diverse plant growth promotion properties such as atmospheric nitrogen fixation, phosphorus solubilization, production of IAAs and siderophores, carbon cycling and plant protection against pathogens.

Since the metabarcoding approach of 16S rRNA and *nifH* genes only allowed us to identify communities that are potentially involved in important processes such as nitrogen fixation, metatranscriptomics analyses are recommended to study gene expression involved in crucial biological processes. Future research will also focus on root-bacteria nodulation and nitrogen-fixation with legumes and respective rhizosphere soils used in this study. If effective, such strains will be characterized and recommended for development as potential inoculum in the agricultural industry.

## Figures and Tables

**Table 1:** Alpha diversity indices determined using the 16S rRNA and *nifH* barcodes for different legume species.

	Alpha Diversity Indices ^1^		
Legume Species	Richness (Observed Taxa)	Shannon	Shannoneven
16S rRNA			
*C. sericea*	546 ± 25.8 ^a^	5.69 ± 0.07 ^a^	0.905 ± 0.005 ^a^
*L. diffusa*	496 ± 34.7 ^a^	5.63 ± 0.09 ^a^	0.908 ± 0.007 ^ab^
*V. karroo*	687 ± 31.6 ^b^	6.09 ± 0.08 ^b^	0.932 ± 0.006 ^b^
*W. monoptera*	482 ± 44.8 ^a^	5.45 ± 0.11 ^a^	0.883 ± 008 ^a^
*nifH*			
*C. sericea*	65.5 ± 10.0 ^a^	2.06± 0.21 ^ab^	0.50 ± 0.04 ^ab^
*L. diffusa*	45.2 ± 13.6 ^a^	1.53 ± 0.28 ^a^	0.40 ± 0.06 ^a^
*V. karroo*	115.0 ± 12.1 ^b^	2.91 ± 0.25 ^b^	0.62 ± 0.05 ^b^
*W. monoptera*	72.9 ± 17.2 ^ab^	2.83 ± 0.36 ^b^	0.67 ± 0.08 ^b^

^1^ Values are means and standard errors for the effect of legumes (*n* = 9; *C. sericea*, *n* = 5; *L. diffusa*, *n* = 6; *V. karroo*, *n* = 3; *W. monoptera*). Different letters (a,b), against the values indicate legume species with significant differences (*p* < 0.05).

**Table 2:** Pearson linear correlation (r) and regression (r^2^) analysis of the Alpha diversity indices with soil physico-chemical properties.

Alpha Diversity Indices		Soil pH	TN [%]	TC [%]	NH_4_^+^ [mg kg^−1^]	NO_3_^−^ [mg kg^−1^]
Richness (Observed taxa)						
16S rRNA	r	0.70 ***	0.54 **	0.52 *	ns	ns
	r^2^	0.46 ***	0.21 *	0.20 *	ns	ns
*nifH*	r	ns	0.62 **	0.66 ***	0.42 *	0.46 *
	r^2^	ns	0.38 **	0.43 ***	0.18 *	0.21 *
Shannon Index						
16S rRNA	r	0.77 ***	0.53 **	0.50 *	ns	ns
	r^2^	0.56 ***	0.22 **	0.18 *	ns	ns
*nifH*	r	ns	0.55 **	0.59 **	ns	ns
	r^2^	ns	0.30 **	0.35 **	ns	ns
Shannoneven Index						
16S rRNA	r	0.79 ***	0.49 *	0.42 *	ns	ns
	r^2^	0.59 ***	0.21 *	0.15 *	ns	ns
*nifH*	r	ns	ns	0.45 *	ns	ns
	r^2^	ns	ns	0.20 *	ns	ns

Abbreviations: TN, total nitrogen; TC, total carbon; NH_4_^+^, ammonium; NO_3_^−^, nitrates. Significance levels: ns, not significant at *p* > 0.05, significant at *p <* 0.05 *; *p* < 0.01 **, and *p* < 0.001 *** levels.

## Supplementary Materials

The following supporting information can be downloaded at: https://www.mdpi.com/article/10.3390/microorganisms10020216/s1, Table S1: Statistical analysis of soil chemical properties of the study sites showing main effects of different legume species and sites, Table S2: Diverse bacterial phyla in the Succulent Karoo biome in South Africa, their inherent properties and possible impacts on plant communities, Table S3: Families containing diazotrophic bacteria (*nifH* gene) in the rhizosphere soils examined and their possible roles in plant growth promotion of legume species in the Succulent Karoo biome in South Africa, Table S4: Analysis of variance (ANOVA) and Permutations analysis of variance (PERMANOVA) to test the effects of factors ‘legume species’, ‘sites’ and their interactions on the respective alpha and beta diversity matrices, using the 16S rRNA and *nifH* gene barcodes.
