# Supplementary material for: Rhizosphere Diazotrophs and Other Bacteria Associated with Native and Encroaching Legumes in the Succulent Karoo Biome in South Africa
Source: Microorganisms. 2022 Jan 20;10(2):216. doi: 10.3390/microorganisms10020216 (PMC8880511; doi:10.3390/microorganisms10020216)
Supplement: Supplementary file 1 [file microorganisms-10-00216-s001.zip › microorganisms-1494017-supplementary.pdf]

Supplementary Information

Diversity and abundance of diazotrophs and other bacteria associated with legumes in the Succulent Karoo biome in South Africa

Esther K. Muema*, Emma T. Steenkamp, Stephanus N. Venter

Department of Biochemistry, Genetics and Microbiology, DSI-NRF Centre of Excellence in Plant Health Biotechnology, Forestry and Agricultural Biotechnology Institute, University of Pretoria, South Africa

***Corresponding author:**

Dr. Esther K. Muema

Phone: +27 (0) 641563314

Email: esther.muema@fabi.up.ac.za

Table caption

Table S1. Statistical analysis of soil chemical properties of the study sites showing main effects of different legume species and sites.

Table S2. Diverse bacterial phyla in the Succulent Karoo biome in South Africa, their inherent properties and possible impacts on plant communities.

Table S3. Families containing diazotrophic bacteria (*nifH* gene) in the rhizosphere soils examined and their possible roles in plant growth promotion of legume species in the Succulent Karoo biome in South Africa.

Table S4. Analysis of variance (ANOVA) and Permutations analysis of variance (PERMANOVA) to test the effects of factors ‘legume species’, ‘sites’ and their interactions on the respective alpha and beta diversity matrices, using the 16S rRNA and *nifH* gene barcodes.

Table S1. Statistical analysis of soil chemical properties of the study sites showing main effects of different legume species and sites.

Factors	Geochemical properties [1, 2]				
	Soil pH	TN [%]	TC [%]	NH_4^+ [mg kg^{-1}]	NO_3^- [mg kg^{-1}]
**Legumes**					
C. sericea	5.08 ± 0.28^a	0.05 ± 0.02^a	0.76 ± 0.26^a	8.67 ± 2.64^a	5.6 ± 2.8^a
L. diffusa	5.46 ± 0.37^{ab}	0.05 ± 0.03^a	0.61 ± 0.35^a	12.68 ± 3.54^a	1.10 ± 0.34^a
V. karroo	6.47 ± 0.34^b	0.16 ± 0.03^a	1.93 ± 0.32^b	17.07 ± 3.23^a	10.87 ±3.65^a
W. monoptera	4.00 ± 0.48^a	0.07 ± 0.04^a	1.08 ± 0.45^{ab}	14.39 ± 4.57^a	0.56 ± 0.22^a
**Sites**					
Kamiesberg	4.67 ± 0.54^a	0.08 ± 0.04^a	1.15 ± 0.47^a	8.97 ± 4.11^a	3.07 ± 1.57^a
Brakputs	5.64 ± 0.27^a	0.09 ± 0.02^a	1.11 ± 0.22^a	13.92 ± 2.05^a	4.65 ± 2.30^a
Kamieskroon	4.97 ± 0.62^a	0.06 ± 0.05^a	0.80 ± 0.54^a	9.51 ± 4.74^a	13.65 ± 5.31^a
**Source of variation and statistical significance [3]**					
Legumes (L)	**	ns	*	ns	ns
Site (S)	ns	ns	ns	ns	ns
L x S	ns	ns	ns	ns	ns

[1] Values are means and standard error for the effect of legumes (n=9; *C. sericea*, n=5; *L. diffusa*, n=6; *V. karroo*, n=3; *W. monoptera*) and sites (n=4; Kamiesberg, n=16 Brakputs, n=3; Kamieskroon). Different letters (a-b), against the values indicate legume species with significant differences ($P < 0.05$).

[2] TC = Total carbon, TN = Total nitrogen, NH_4^+ = Ammonium, NO_3^- = Nitrates.

[3] Significance levels: ns = not significant at $P > 0.05$, significant at $P < 0.05$ * and $P < 0.01$ ** levels.

Table S2. Diverse bacterial phyla in the Succulent Karoo biome in South Africa, their inherent properties and possible impacts on plant communities.

Phylum	Percent of total sequences	Inherent properties	Plant impacts	References
Actinobacteria	26.7%	Heat and soil acidity tolerant	Carbon nutrient provision through decomposition and mineralization of soil organic matter	Makhalanyane et al., 2015
Proteobacteria	23.5%	Nutrients limited arid soils adapted.	Plant growth promotion through biological nitrogen fixation	Andrews and Andrews, 2017
			Antagonistic effects against plant pathogens	Ofek et al., 2012
Acidobacteria	10%	Adapted to acidic and nutrients poor environments	Plant growth promotion through production of indole acetic acids (IAA) and siderophores	Op den Camp et al., 2009; Kielak et al., 2016;
Planctomycetes	10%	Adapted to extreme temperatures and pH	Plant growth promotion through provision of carbon via carbon metabolism.	Kovaleva et al., 2015.
			Plant growth promotion through ammonia oxidation in oxygen limited conditions to nitrates	Strous et al., 1999
Chloroflexi	6.3%	Adapted to extremely hot environments	Provision of carbon nutrient through degradation of complex organic compounds	Xia et al., 2008

Table S2. Continued.

Bacteroidetes	7.6%	Adapted to extreme temperatures	Carbon cycling through degradation of complex organic compounds.	Xia et al., 2008; Jorquera et al., 2012
			Promotion of plant growth through nitrogen fixation.	Makhalanyane et al., 2015
Verrucomicrobia	7.3%	Adapted to acidic, high temperature and nutrients poor environments	Plant growth promotion through nitrogen fixation and carbon cycling	Khadem et al., 2010
			Provision of organic carbon to plants.	Op dem Camp et al., 2009
Cyanobacteria	0.8%	Can withstand desiccation, high temperatures and unstable pH	Plant growth promotion through fixation of atmospheric nitrogen into ammonium and nitrates	Dojani et al., 2014;
Gemmatimonadetes	1.8%	Adapted to extremely hot and moisture limited environments	Plant growth promotion through photosynthesis	Zeng et al., 2017; Makhalanyane et al., 2015
Firmicutes	0.9%	Adapted to extremely hot and dry environments	Plant growth promotion via phosphorus solubilization, nitrogen fixation, production of IAAs, siderophores, photosynthesis and antagonistic effects against plants pathogens	Felestrino et al., 2017; Jorquera et al., 2012

Table S3. Families containing diazotrophic bacteria (*nifH* gene) in the rhizosphere soils examined and their possible roles in plant growth promotion of legume species in the Succulent Karoo biome in South Africa.

Family	Percent of total sequences	Legume rhizosphere soil	Role in rhizosphere/soil	References
Bradyrhizobiaceae	4.6%	*C. sericea, W. monoptera, V. karroo*	Plant growth promotion through biological nitrogen fixation	Andrews and Andrews, 2017; Moiloa, 2016
Rhizobiaceae	4.2%	*C. sericea, L. diffusa, V. karroo*	Plant growth promotion through biological nitrogen fixation	Andrews and Andrews, 2017; Räsänen et al., 2001; Sankhla et al., 2017
Nostocaceae	24.4%	*C. sericea, W. monoptera, L. diffusa, V. karroo*	Plant growth promotion through biological nitrogen fixation	Dojani et al., 2014
Comamonadaceae	0.18%	*C. sericea, V. karroo*	Promotion of growth through nitrogen fixation and carbon cycling	Schmalenberger et al., 2008
Phyllobacteriaceae	39.0%	*C. sericea, L. diffusa, V. karroo*	Plant growth promotion through biological nitrogen fixation	Andrews and Andrews, 2017; Phalane et al., 2008; Gerding et al., 2012
Microchaetaceae	4.0%	*W. monoptera, V. karroo*	Plant growth promotion through biological nitrogen fixation	Dojani et al., 2014
Rhodocyclaceae	3.7%	*C. sericea, V. karroo*	Free nitrogen fixation	Bae et al., 2007

Table S3. Continued.

Rhodospirillaceae	7.9%	*C. sericea, V. karroo, W. monoptera*	Plant growth promotion through nitrogen fixation	Bao et al., 2013; Selao, 2010
Methylobacteriaceae	3.0%	*C. sericea, V. karroo, W. monoptera*	Plant growth promotion through biological nitrogen fixation	Moiloa, 2016
Acetobacteraceae	1.1%	*C. sericea, V. karroo, W. monoptera*	Plant growth promotion through phosphate solubilization, nitrogen fixation and plant pathogen control	Reis and Teixeira, 2015
Scytonemataceae	3.1%	*L. diffusa, W. monoptera*	Plant growth promotion through biological nitrogen fixation	Dojani et al., 2013
Sphingomonadaceae	0.12%	*C. sericea, V. karroo*	Plant growth promotion through production of Indole acetic acids	Tsavkelova et al., 2007;
Enterobacteriaceae	0.06%	*W. monoptera*	Plant growth promotion through free-living biological nitrogen fixation	Schmitz et al., 2002
Geobacteraceae	1.78%	*V. karroo*	Plant growth promotion via nitrogen fixation	Berthrong et al., 2014; Holmes et al., 2004

Table S3. Continued.

Desulfovibrionaceae	0.62%	*V. karroo*	Plant growth promotion via nitrogen fixation	Zhang et al., 2017; Bertics et al., 2013
Ectothiorhodospiraceae	0.16%	*V. karroo*	Capacity for plant growth promotion through nitrogen fixation	Tourova et al., 2007
Lachnospiraceae	0.20%	*V. karroo*	Plant growth promotion through nitrogen fixation	Sarria-Guzmán et al., 2016
Pseudomonadaceae	2.7%	*V. karroo*	Plant growth promotion through P solubilization, N fixation, production of siderophores for iron transport to plants and plant pathogens control	Song et al., 2021
Alcaligenaceae	1.2%	*V. karroo*	Plant growth promotion through nitrogen fixation and production of Indole acetic acid	Pedersen et al., 2018; Panke-Buisse et al., 2015.

Table S4. Analysis of variance (ANOVA) and Permutations analysis of variance (PERMANOVA) to test the effects of factors 'legume species', 'sites' and their interactions on the respective alpha and beta diversity matrices, using the 16S rRNA and *nifH* gene barcodes.

**Diversity measures**	**Effects/sources of variation** [1]		
	**Site**	**Legumes**	**Site x Legumes**
**Alpha diversity (ANOVA)**			
16S rRNA			
Richness (observed taxa)	ns	**	ns
Shannon diversity	ns	***	ns
Shannon evenness	ns	***	ns
nifH			
Richness (observed taxa)	ns	**	ns
Shannon diversity	ns	**	ns
Shannon evenness	ns	*	ns
**Beta diversity (PERMANOVA)**			
16S rRNA			
Bray-Curtis	ns	***	ns
Jaccard	ns	***	ns
nifH			
Bray-Curtis	**	**	ns
Jaccard	***	***	ns

[1] Significance levels: ns = not significant at $P > 0.05$, significant at $P < 0.05$ *; $P < 0.01$ ** and $P < 0.001$ *** levels.
